# How do people with COPD experience telerehabilitation using exergaming? A qualitative study

**DOI:** 10.1186/s12890-026-04214-1

**Published:** 2026-03-04

**Authors:** Jenny Danielsbacka, Wetter Edvin Diskin, Lowie E.G.W. Vanfleteren, Maria Bäck

**Affiliations:** 1 Department of Occupational Therapy and Physiotherapy, Sahlgrenska University Hospital, Gothenburg, Sweden; 2 Department of Physiotherapy, Institute of Neuroscience and Physiology, Sahlgrenska Academy, University of Gothenburg, Gothenburg, Sweden; 3 COPD Centre, Department of Respiratory Medicine and Allergology, Sahlgrenska University Hospital, Gothenburg, Sweden; 4 Department of Internal Medicine and Clinical Nutrition, Institute of Medicine, Sahlgrenska Academy, University of Gothenburg, Gothenburg, Sweden; 5 Department of Respiratory Medicine, Ghent University Hospital, Ghent, Belgium; 6 Department of Molecular and Clinical Medicine, Institute of Medicine, Sahlgrenska Academy, University of Gothenburg, Gothenburg, Sweden; 7 Department of Physical Therapy, Sahlgrenska University Hospital, Vita stråket 13, Gothenburg, SE 431 45 Sweden

**Keywords:** COPD, Pulmonary Rehabilitation, Exergaming, Tele monitoring, Qualitative research

## Abstract

**Background:**

Centre-based pulmonary rehabilitation improves symptoms and exercise tolerance in patients with chronic obstructive pulmonary disease (COPD), but barriers such as accessibility and adherence limit participation. Home-based pulmonary telerehabilitation (PTR) may provide a feasible alternative, but patient perspectives are not well understood. This study explored the experiences and perceptions of patients with COPD regarding participation in a home-based PTR, using an exergaming system.

**Methods:**

This qualitative study design used individual semi-structured interviews with 11 patients with COPD who had completed a 12-week exergaming PTR programme. The interviews were transcribed verbatim and analysed via inductive content analysis, as outlined by Graneheim and Lundman.

**Results:**

An overarching theme was identified as “well-functioning technology at home, with continuous support from a physiotherapist, increases the opportunities for people with COPD to exercise on their own individual terms”. Three major categories and nine subcategories captured participants’ experiences. The first major category, “technology and equipment challenge and enable exercise at home”, captured experiences of flexibility, playful feedback, and motivation, but also frustration with technical issues and limited space. The second main category, “Getting the right support adds value when exercising with exergaming”, included reassurance, motivation, and enhanced self-efficacy through physiotherapist feedback, monitoring, and individual adaptation. The third category, “there are considerable challenges for people with COPD regarding exercise”, encompassed symptom variability, the need to balance effort with daily capacity, and mixed experiences of competitive elements, although some participants reported functional improvements and reduced fear of exertion.

**Conclusion:**

Home-based exergaming PTR was acceptable and motivating when the technology was reliable and physiotherapist support was sustained. Programmes should prioritise robust equipment, clear feedback, and individualised exercise to enhance adherence and long-term benefit.

**Trial registration:**

The study is registered in the database FOU i Sverige (R&D in Sweden) with registration number: 273,768, date for registration: April 1, 2020.

**Supplementary Information:**

The online version contains supplementary material available at 10.1186/s12890-026-04214-1.

## Background

Chronic Obstructive Pulmonary Disease (COPD) is a prevalent and progressive lung disease caused by prolonged exposure to noxious particles or gases, particularly through habitual tobacco smoking [1]. Patients with COPD typically suffer from chronic respiratory symptoms, reduced exercise capacity, and diminished health-related quality of life [2].

Exercise training as part of pulmonary rehabilitation (PR) is a cornerstone of COPD management and is recommended in international guidelines [2]. PR programmes consistently demonstrate improvements in symptoms, exercise tolerance prognosis and healthcare utilisation [2] Despite strong evidence supporting their benefits, access to and adherence with organised centre-based exercise programmes remain limited, posing a significant barrier to effective implementation [2]. Moreover, high-quality exercise programmes require individualisation based on each patient’s baseline assessment and ongoing adaptation according to their progress. The effectiveness of exercise programmes depend largely on the degree of guidance, coaching, and supervision from dedicated physiotherapists [3, 4].

To overcome these barriers, home-based pulmonary telerehabilitation (PTR) has emerged as a promising alternative. PTR offers greater accessibility and has been increasingly implemented in clinical practice [5]. A recent meta-analysis showed that PTR for people with chronic respiratory disease achieves outcomes comparable to those of traditional centre-based PR, with no safety concerns [6]. Nevertheless, technological approaches to PTR vary widely, and standardised protocols are lacking. Our research group developed a home-based exergaming system for the present study. The system aims to provide appropriate structured and high-quality guideline-based PTR. Evaluating patients´ experiences with such systems is of crucial importance for optimising their design and implementation.

### Aim

The aim of this study was to explore patients´ experiences and perceptions of participating in a home-based exercise using the exergaming system. An increased understanding of these experiences will inform the continuing development of exergaming interventions for patients with COPD.

## Methods

### Design

This study is a qualitative interview study where individual semi-structured interviews were conducted with the participants. Analysis was conducted using an inductive, descriptive approach through qualitative content analysis, as outlined by Graneheim and Lundman [7]. Two theoretical assumptions guided this qualitative content analysis method. First, interviews were shaped through interaction between the participant and the researcher, since ‘one cannot not communicate’. Second, reality can be interpreted in various ways, with understanding depending on researchers’ subjective interpretation [7]. The interpretation is always influenced by the researchers’ pre-understanding, meaning that the experiences and qualifications the researcher have is important to acknowledge [7]. The inductive approach implies that the analysis is data-driven and characterised by researchers seeking patterns within the text [8].

### Materials and setting

The participants included in the study were all part of the intervention group in the randomised controlled trial (RCT), named “Feasibility and effectiveness of a home monitored rehabilitation intervention using an exergaming approach—a randomised, controlled clinical study” at the COPD Centre in Sahlgrenska University Hospital (clinical trials ID: NCT04816825). For the study a home-based exergaming system was developed. The system combined: (1) game-console based exercise programmes, (2) exercises specifically designed by professional physiotherapists for patients with a chronic lung disease, and (3) remote monitoring with close supervision. All participants used a study-provided portable Wi-Fi connection to enable stable access to the exergaming system and telemonitoring functions.

All participants had moderate to very severe COPD and were prescribed the home-based exercise programme using an PTR system designed by Alkit communications AB in collaboration with the Sahlgrenska COPD Centre.

### Intervention

The intervention was 12 weeks long, consisting of up to 60 min of aerobic and resistance training three times per week. Each session featured up to 20 min of gamified aerobic exercise on a mini-bike as well as guided strength exercises using resistance bands through movement recognition and automated movement quality feedback. The system was connected to a backend server, enabling physiotherapists to telemonitor participants’ activity and exercise adherence. Participants received weekly feedback and individualised progression of their exercise programme via telephone.

Eligibility criteria for this qualitative study included completion of the 12-week rehabilitation period in the RCT and the ability to be interviewed within 2 months of finishing the RCT. The inclusion period for the qualitative study took place during the last eight months of the RCT; from March 4, 2024, to October 14, 2024. At the time of the final assessment in the RCT, participants were asked by the research physiotherapist (EDW) whether a researcher (JD) could contact them regarding the qualitative interview study. At that time, participants also received written information about the interview study. Twelve people were approached regarding participation. One declined to participate after receiving the study information, without providing a reason, resulting in eleven participants being included. All participants gave their written informed consent before inclusion. The interview guide was developed for this study and has not previously been published elsewhere. The Ethics Review Board of Gothenburg approved the study (Ref number: 2020–02292) The study is registered in the database “FOU i Sverige” (R&D in Sweden) with registration number: 273,768.

### Procedure

A researcher (JD), who had no previous relationship with the participants, conducted the interviews. She presented herself to the participants as a researcher at Sahlgrenska University Hospital. The participants could choose to be interviewed at home, at Sahlgrenska University Hospital, via digital meeting on Zoom, or by telephone. One interview per participant was performed. A semi-structured interview guide (Supplementary file 1) was used comprised of open-ended questions covering several topics. All interviews began with the open-ended question: “Can you tell us about your experiences training with the exergaming system?” No field notes were taken during the interviews. The resulting sound files were used as the unit of analysis after verbatim transcription. The transcripts were not returned to participants for correction, comment, or feedback. In qualitative research, sufficient sample size depends on the quality and richness of the data, and it is not possible to predetermine a specific number of required interviews [8, 9]. In the present study, all eleven interviews were performed before commencing analysis. After completing the analysis process, no new topics appeared, which indicates that adequate data for being able to answer the research questions was reached.

### Analysis

Qualitative latent content analysis according to Graneheim and Lundman [7] was used, and analysis was performed in several steps; see Table 1. The analysis followed the guidelines outlined by Malterud [10] and the Standards of Reporting Qualitative Research (SRQR) [11] to ensure the trustworthiness of the analytical process. Interviews were written out verbatim upon completion, listened and read through several times to get a sense of the entirety of the texts. The analysis was carried out by searching for meaning units in the text that corresponded to the aim of the study. The meaning units were then condensed and classified with codes. The final stage of the analysis was to sort the codes into categories and sub-categories. During analysis, researchers continuously moved between the entire text and its parts to ensure that the interpretation reflected the participants’ experiences as credibly as possible. Authors JD, EDW and MB conducted simultaneous analyses of the interviews initially and discussed the results throughout the process. Finally, the results were triangulated to ensure credibility and transparency of the analysis, meaning that the preliminary subcategories and categories were discussed between all authors until consensus was reached. Three of the authors are physiotherapists (JD, EDW, MB) and one is a physician (LGEWV). Two of the authors are women with experience in the field of qualitative research (JD, MB). All authors had a pre-understanding through clinical experience working with COPD, and EDV and LGEWV had experience with the exergaming system.

**Table 1:** The analysis process of qualitative content analysis according to Graneheim and Lundman

1. The interviews were listened and read through several times to create a sense of the whole.
2. Topics corresponding to the aim of the study were marked in the interviews, i.e. the unit of analysis.
3. The marked text were divided into meaning units.
4. The meaning units were condensed, i.e., the sentences were shortened without changing the meaning.
5. The condensed text was abstracted which means that the content and interpretation of the condensed text was described at a higher level of abstraction in a code.
6. These codes were then sorted based on similarities and differences. The codes were divided into categories and sub-categories.

## Results

Eleven participants—six women and five men—with moderate to very severe COPD were included in the study. Table 2 summarises their demographics and clinical characteristics.

**Table 2:** Characteristics of the study participants (n: 11)

Demographics	
Age, y	71 (58, 82)
Women/Men, n/n (%/%)	6/5 (54.5/45.5)
FEV1, % of predicted	37 (24, 78)
GOLD group B, n (%)	7 (63.6)
GOLD group E, n (%)	4 (36.4)
COPD Assessment Test (CAT) score	22 (12, 29)
6 min walk test, m	320 (240, 495)

*6MWT* 6-minute walk test, *CAT* COPD Assessment test, *FEV* GOLD, Global initiative for Chronic Obstructive Lung disease, *FEV1* Forced Expiratory Volume in one second

Data presented as median (min, max) unless otherwise stated

Two interviews were conducted through video connection and nine were held via telephone. The interviews were recorded and had a median duration of 27 min (min 18 min, max 52 min).

**Fig. 1:**
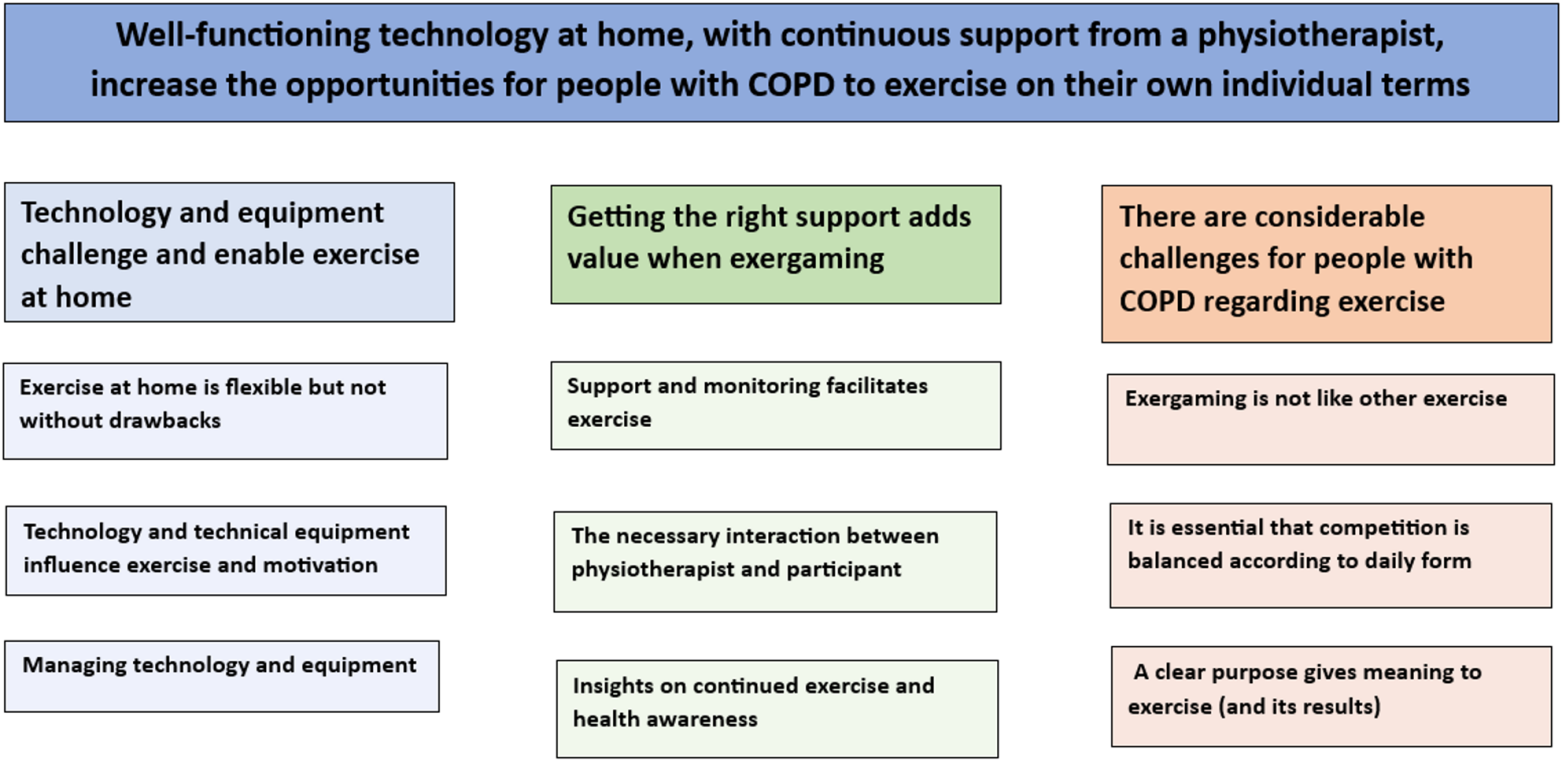
Theme, major categories and sub-categories identified in the study

An overarching theme was identified in the unit of analysis: “well-functioning technology at home, and continuous support from a physiotherapist increase opportunities for people with COPD to exercise on their own individual terms.” Within this theme, three major categories were identified: “technology and equipment challenge and enable exercise at home”, “getting the right support adds value when exercise with exergaming” and “there are considerable challenges for people with COPD regarding exercise.” All three major categories contain a number of sub-categories; see Fig. 1.

### Theme: well-functioning technology at home, with continuous support from a physiotherapist, increase opportunities for people with COPD to exercise on their own individual terms

This overall theme captures different perspectives of the participants’ experiences and perceptions of exercising with the exergaming system in their homes. For participants, it was of great importance that the technological aspects of the system, as well as the technical equipment, functioned reliably to support their exercise. When technical difficulties occurred, they often led to frustration and reduced motivation. The importance of the role of the physiotherapist role in creating a safe, professional, and individually prescribed and adapted exercise environment was identified. These two aspects—well-functioning technology and continuous professional support—were highlighted by participants as key facilitators of exercise.

### Major category 1: technology and equipment challenge and enable exercise at home

In the first major category, both the advantages and disadvantages of exercising at home are described. Perspectives ranging from practical matters, such as having enough space at home for equipment, to personal aspects, including safety and accessibility, were highlighted. Technology and supporting equipment were acknowledged not only as tools that facilitate exercise at home, but also as potential sources of frustration in cases where they were not working sufficiently. This matter was, however, described as manageable through caregiver support, as well as empowering when one was able to handle the equipment independently after receiving instruction. The major category includes the following sub-categories:

#### Subcategory 1.1: exercise at home is flexible, but not without drawbacks

Participants highlighted the flexibility of exercising at home with the exergaming system. The fact that the system could be used to perform an exercise session divided into several shorter bouts during the day was seen as an advantage, as participants might not always be able to complete a full session at once. The sessions were scheduled for specific days but could be shifted slightly, which gave participants the confidence needed to complete their exercise despite unforeseen events.

Sometimes I did it in the morning and sometimes I did it late at night. Well, it was a big advantage to have it at home.

Participant 9.

Exercising at home was mostly seen as advantageous; nonetheless there were some practical questions connected to the exergaming equipment, such as whether it fit in the home and how to store it between sessions.

The only downside was that you couldn’t keep it upright and had to pick it up all the time. That was the only negative thing if I say so myself.

Participant 8.

Positive aspects of exercising at home included the removal of comparisons with others, easy accessibility, time savings, and not having to change into training clothes, all of which made it difficult to find an excuse and supported exercise adherence.

That’s the advantage that you can do it at any time, whenever you want…for example, now that we’ve finished talking, I can go out and take a class without having to plan it.

Participant 3.

It was also easy to create a sense of security around exercise by planning to have inhalers and/or water close at hand. However, missing out on social interaction with others when exercising at home was highlighted as a disadvantage.

I always had water and also my Buventol next to me, like psychological security.

Participant 8.

#### Subcategory 1.2: technology and technical equipment influence exercise and motivation

Different perspectives regarding the technology and exercise equipment that affected their ability and motivation to exercise were raised by the participants. Regular system updates could disrupt planned exercise sessions and sometimes led to participants skipping exercise that day. Equipment, such as the exercise bike and the finger probe for the pulse oximeter, also led to frustration when they were unstable or malfunctioning, and broken equipment was raised as a large disadvantage.

So there were a few times that I gave up on riding my bike for the third time because I just felt like I couldn’t keep up. And I sat there turning this steering wheel back and forth, back and forth and nothing happened.

Participant 1.

Unsuccessful blood oxygen saturation and pulse measurements ​​were considered troublesome, as they created uncertainty about how the exercise actually went, but also provided security when obtaining correct values. It was mentioned that without correct measurements, the exercise felt pointless, but when registered correctly, it strengthened participants’ belief in the utility of the exercise and provided structure.

Well, it was a bit interesting, but I can’t really tell what it is, whether it was good or bad at the moment.

Participant 7.

Seeing yourself in the animated computer environment was considered positive, because it gave the exercise a sense of playfulness and made exercising feel fun. Activating the eye through the images was seen as positive and increased participants’ motivation to train.

Well, it was very cool. Yes, you could see yourself in the picture there, working out and yes… it made it a little more fun and stuff.

Participant 10.

#### Subcategory 1.3: managing technology and equipment

The experiences of support regarding technology and technical equipment varied. While some participants felt that support was provided quickly, others described delays that disrupted their exercise. Support was mainly thought of as easily accessible and provided a sense of security.

And so I always had their phone number, he said, “If there’s anything, just call…” It felt safe to know that you can call someone and ask for advice or help or solve the problem. It felt safe to know that you can do this.

Participant 6.

Receiving support on how to manage the equipment yourself if it malfunctioned was considered positive. Experiences varied regarding the sufficiency of the instructions given to handle the device before intervention. It was perceived as positive that the pre-intervention instructions were given in several ways, both verbally and in writing. This created a sense of safety and preparedness before the start.

It was absolutely excellent, because I came to Sahlgrenska…and then the physiotherapist came down and met me…and then we went through the equipment and I got to test the computer and try the exercises, so that I felt really confident in it, really.

Participant 2.

The participants also felt that the system itself was easy to use, although some experienced anxiety before starting regarding their ability to handle the technology. The system was considered user-friendly both in terms of using the computer itself (starting the system and entering the program) and in terms of performing the exercise (changing exercises, doing your exercise session).

I didn’t think it was that complicated even though I’m an old man, since I got into it pretty quickly I thought, so it wasn’t that complicated I thought.

Participant 7.

An advantage of the system experienced by participants was that it provided feedback if exercises were performed incorrectly, and that there was a countdown preceding every exercise, making them easier to perform.

It was fun because you could also follow when you were finished, well, you could see how many you were doing all the time, you could follow everything. It felt good.

Participant 4.

### Major category 2: getting the right support adds value when exergaming

The second major category highlights how participation in a study enhances motivation and provides a sense of purpose, supporting the continuation of exercise despite challenges.

The role of the physiotherapist as a mentor and expert in COPD management was perceived as both safe and as offering assurance, and additionally as absolutely necessary for keeping up with the exercise. Continued support from the physiotherapist, coupled with improved understanding of the role of exercise in COPD management led to changes in perception regarding exercise and future self-care. The major category comprises the following sub-categories:

#### Subcategory 2.1: support and monitoring facilitate exercise

Participants agreed that being part of the study made exercising easier than it would have been otherwise. Knowing that their exercise was being monitored meant that they could not avoid exercising or trying to cheat during an exercise session.

But it was a good way to train, because you couldn’t skip training and you still had to complete what you had committed to.

Participant 10.

Receiving support about both the system and the exercise itself, was seen as positive. Participants highlighted that the structure of exercise was important for it to be carried out, and the monitoring aspect could be seen as a reminder to keep going and to ensure that the exercise was carried out.

I myself have thought that yes, we have these kinds of training things that are on TV and stuff and so on, but it has never happened that you start….

Participant 8.

Being monitored was not seen as a disadvantage, but rather something that instilled a feeling of safety. Knowing someone was monitoring their data was motivating and allowed the participants to exert themselves more thoroughly during exercise without having to be afraid.

And the physiotherapist kept track of my pulse and my oxygen saturation, the whole thing, he then checked it after I reported it to him so he could see the values.

Participant 6.

#### Subcategory 2.2: the necessary interaction between physiotherapist and participant

The physiotherapist played a major role in making the participants feel supported and seen regarding their exercise and their well-being during the study, and this was carefully pointed out by the participants. The physiotherapist was considered a mentor who provided both practical and emotional support regarding exercise.

He was very calm and supportive, I must say. It was very, I felt very safe with him, I actually did.

Participant 5.

Receiving fact-based, competent advice from the physiotherapist provided a sense of security and reduced exercise-related fear and anxiety among the participants. The planned weekly contact with the physiotherapist during the intervention provided security. The exercise programme was individually adapted based on participants’ baseline results and adjusted throughout the study based on their exercise results, which made the exercise feel meaningful.

I want to say, yes, but it is absolutely necessary (the role of the physiotherapist). It is not that you could just get a computer and run it yourself, but it is really important that you meet him, you do this walking training, he gets a status on me beforehand and based on that he can also use his expertise to set up a programme that suits me.

Participant 2.

The physiotherapist was perceived as empathetic and attentive, making sure exercise adaptations were performed in agreement with the participants. This allowed participants to experience that their exercise was adapted on their own terms.

I think it’s good that it was individualised so that you could—that they did exercises according to your ability, you could more or less say.

Participant 1.

#### Subcategory 2.3: insights on continued exercise and health awareness

Exercising with the exergaming system led some participants to change their views on exercise, and in several cases, induced a newfound desire to take responsibility for their own well-being going forward. Participating in the study stimulated new exercise habits and gave participants increased knowledge regarding how they could exercise safely, despite their COPD diagnosis.

That’s been my thought after this adventure to try to maintain. Because when I sat with him (the physiotherapist) after that evaluation, then it was quite, well, it was, it felt *with tears in the voice* I was so affected that there was such a marked difference.

Participant 8.

However, it was also expressed that participants had a desire to increase their exercise, but that it was difficult to carry out exercise outside the home for both practical and financial reasons. Participants expressed increased general health awareness and said that they had become more aware of their diet and also reduced their alcohol intake. Importantly, it underscored the role of exercising when living with COPD.

I need to get a little better, I think I’m getting worse with COPD and there’s only one thing that helps and that’s exercise.

Participant 11.

### Major category 3: there are considerable challenges for people with COPD regarding exercise

In the third major category, participants compared their previous exercise experiences with exergaming, leading some to dwell on their physical activities levels prior to being diagnosed with COPD. The perceived importance of balancing activity levels with energy levels was highlighted, as were the rejuvenating effect of physical activity. Goal-oriented exercise, aimed at improving functions impaired by COPD, was emphasised as particularly meaningful.

This major category comprises the sub-categories:

#### Subcategory 3.1: exergaming is not like other exercise

The participants felt that exercise via the exergaming system was difficult to compare to other exercise. According to them, the exercise they had done before receiving their COPD diagnosis was on a completely different level and nothing that they could perform in the present.

I can’t compare it. It won’t be the same at all. Not for me. There you went to…what’s it called, you rowed, and you skied, and you walked on a treadmill, and lifted scrap. It’s not the same thing. And I absolutely can’t do that since I got sick then, it doesn’t work for me. So, for me it’s not possible to compare.

Participant 4.

Calling exergaming “a game” was something participants did not agree with, as the purpose of a game is just to have fun, while exergaming was about rehabilitation exercise aimed at reducing their COPD symptoms. Participants perceived that the equipment differed from what is found in a gym, and that it was difficult to compare it with other forms of exercise; especially for those with little prior training experience.

I don’t usually get muscle aches when I’m sitting and playing games on the computer or on the tablet or on the phone, like you get when you do these exercises. So I don’t think I call this a game.

Participant 1.

#### Subcategory 3.2: it is essential that competition is balanced according to daily form

The fact that the exergaming system had built-in competitive elements, such as showing previous times for completing an exercise and presenting audiovisual feedback when records were broken, sparked many different thoughts among the participants. For some, it was just a fun feature that encouraged them to try to get better with each exercise session, and for others it sparked their competitive spirit and encouraged them to break earlier records.

That you have something to look forward to, that you have something to, well, compete with yourself and try to get better all the time, in the exercises that you do. In terms of time and that you will have more energy all the time, that was actually good.

Participant 6.

For others, trying to beat previous records was unhelpful, as it could prevent them from completing the whole exercise session. This became a balancing act, and a challenge to try to find suitable levels of exercise that were adapted to your own current state.

Yes, I didn’t compete that way, maybe. Yes, I saw what my best results were and tried hard, but yes. It really depended on my form that day too.

Participant 7.

Learning to trust your body and its signals, understanding what you could manage on a given day, or daring to exercise even when you were not feeling at your best, became a decision made on a session-by-session basis. Having to adapt to daily functional limitations was considered frustrating, and it became important for participants to think they were doing the best they could and that they were exercising for their own benefit, rather than for external validation.

Because it’s also part of learning about your own body. You can’t just rely on others to tell you how, what makes you feel good and what doesn’t. You have to feel that yourself when you have this disease.

Participant 2.

#### Subcategory 3.3: a clear purpose gives meaning to exercise (and its results)

It was important for participants that the purpose of the exercises was clear. Knowing why an exercise was important and what it targeted, increased motivation to carry it out, while unclear exercises were experienced as meaningless, which did not yield results.

The most important thing was catching the balloons. It was awesome, because it meant getting up from the chair. It was one of the basic exercises that had been explained to me before, what a difference such exercises make in everyday life. So, I know I took it very seriously, it was really tiring.

Participant 8.

The results of participating in the study varied. Some participants experienced clear improvements to perform everyday activities that had previously been difficult, such as climbing stairs. For others, that experience did not exist, and the feeling that it was difficult to transfer the effect of exercise was present, and there were also concerns that the 12-week exercise period was too short to see lasting changes.

I had difficulty walking and my legs were very tired before, so I had difficulty walking up stairs and such. But even today I still have that, so I can handle stairs very well.

Participant 9.

A psychological effect described by participants was the feeling of being more confident and less fearful of physical activity than before. Seeing clear improvements at the follow-up test after the intervention was seen as positive and encouraging.

It felt good and it also meant that, I’ve always been a little scared before, I’ve always been a little scared that I’d get carried away if you say so, but I’m not anymore.

Participant 4.

## Discussion

This qualitative study is based on interviews with 11 patients with COPD that performed a 12-week PTR intervention using specifically developed interactive supportive symptoms with an exergaming approach. It provided key insights into PTR interventions for patients with COPD. The results indicate that exercising with a COPD diagnosis was challenging, and required both mental and physical adaptation, as well as facilitating circumstances such as sufficient space and well-functioning technology to enable participation. These findings highlight that while home-based telerehabilitation may increase accessibility for some patients, it may also risk exacerbating intervention inequalities, as not all patients have access to adequate living space, reliable internet connectivity, or technical support. This has important implications for the clinical feasibility and equitable implementation of telerehabilitation programmes. The participants found that being part of the current study helped them establish a routine which facilitated structured exercise delivered through exergaming and provided knowledge about exercise and COPD. This in turn increased participants’ self-efficacy towards exercise. Both these factors contributed to reducing participants’ fear of respiratory symptoms and of exercise itself. Moreover, understanding the purpose of the exercise was highlighted as an important factor in increasing motivation. These findings are consistent by those reported by Robinson et al. [12], who identified facilitators of and barriers to exercise following a pulmonary rehabilitation in patients with COPD, including clear exercise intentions, routines, and exercise-related self-efficacy. This indicates that participation in an exergaming programme, such as in the current study, which includes features such as learning about physical activity, and enhance self-efficacy, may contribute to behaviour change beyond the structured rehabilitation context in this patient group.

Although behaviour change techniques were not used as an analytic framework in the present study, several such components were embedded in the intervention design and were highlighted by participants as important for their exercise experience. For example, real-time feedback, performance monitoring, and graded challenges were integral parts of the exergaming system, and participants described these features as motivating and as providing structure and meaning to their exercise. Similarly, continuous interaction with the physiotherapist functioned as professional and emotional support, which participants perceived as essential for maintaining exercise over time. From a behavioural medicine perspective, interventions that incorporate self-monitoring, goal setting, and feedback have been shown to be more effective in increasing physical activity among healthy adults compared to interventions lacking these components, and these principles are increasingly applied to structured exercise and rehabilitation contexts [13]. These strategies are particularly relevant for patients with COPD, for whom motivation, confidence, and long-term adherence to exercise often pose challenges. Chen et al. [14] has shown that applying behavioural change theories in pulmonary rehabilitation can help tailor interventions to patients’ readiness, motivation, and perceived barriers. Such strategies may enhance adherence during rehabilitation and support long-term exercise routines. Recent evidence also highlights that the behavioural change components embedded in gamification are key drivers in their effectiveness. A systematic review found that real-time feedback, adaptive challenges, and personalised goals increased adherence and motivation, with particularly high engagement in virtual reality and exergame-based interventions [15]. These behavioural components were associated with improvements in COPD management, including greater exercise tolerance, improved self-management, and reduced symptom burden. In the present study, participants stated that weekly interaction with the physiotherapist to discuss and adapt the intervention was necessary to increase motivation for exercise and to promote a sense of security. Similarly, Lee et al. [16] found that features such as feedback, motivation, and social interaction positively influenced the use of technology. This indicates that the sole use of supportive technology alone, such as exergaming, is insufficient, and that participants benefit more when the intervention is complemented by interaction with healthcare professionals, such as physiotherapists. Participation in the present study also increased participants´ awareness of their own responsibility for managing their health and continuing exercise after the study participation. According to the European Respiratory Society [17], a key factor in enhancing motivation to engage in physical activity is the provision of performance feedback and the development of action plans, with the aim of increasing opportunities for patients with COPD to engage more in physically activity. This further emphasises the importance of interaction between healthcare professionals and patients engaging in TPR programmes, such as the one investigated in this analysis.

The participants in this study reported varied experiences of the technology and technical equipment used in the exergaming intervention. Overall, the findings indicate that the technology strongly influenced both exercise performance and motivation. By learning to manage the technology independently, with initial or continuing support, and having the opportunity to try the equipment together with the physiotherapist before commencing the intervention, participants felt more confident in using the exergaming system. This suggests an increase in self-efficacy related to the technological aspects of the exercise and is consistent with findings reported by Lee et al. [16], who showed that providing opportunities to practise with the technology before the intervention allowed participants to become familiar with the equipment and increased their engagement. When initiating exercise through technology-based equipment, it is therefore important to pay attention to the introduction process, as this appears to facilitate engagement and support the development of sustainable exercise routines.

### Strengths and limitations

In qualitative research, trustworthiness is achieved through the presentation of credibility, dependability, confirmability, and transferability of the research process as a whole, as well as of the results [7, 11, 18]. Including the characteristics and reflexivity of the researchers when conducting research also augments trustworthiness [9]. The use of recommendations and reporting guidelines [10, 11] for qualitative research in the present study enhances its credibility. In addition, the variation in age, gender and severity of COPD among the participants supports a broad presentation of experiences. A potential limitation is that all participants were recruited from an exercise study, which may indicate a pre-existing positive attitude towards exercise and rehabilitation. However, the aim of the present interview study was to explore experiences of the exergaming system and its perceived advantages and disadvantages, rather than attitudes towards exercise per se. Social and contextual characteristics such as socioeconomic status, social support, housing, and rural/urban location were not collected, as these factors were outside the original scope of the study. This may limit the contextual interpretation and transferability of the findings. Regarding dependability and confirmability, the use of a semi-structured interview guide for all participants and the fact that all interviews were conducted by the same researcher (JD) supported consistency in data collection. In addition, the use of Graneheim’s and Lundman’s [7] analytical process (see Table 1), contributed to a systematic and transparent analysis of the data. All interviews were conducted either via video connection or by telephone, depending on the participants’ preferences. This could be considered a limitation, as the lack of in-person contact may have influenced the depth or richness of the data. However, video-based interviews allowed visual interaction between the participant and the interviewer while still reducing the need for physical presence. In contrast, telephone interviews lack visual cues, making it particularly important that interview questions are clearly formulated and that the interviewer is able to identify and clarify potential misunderstandings during the interview. The use of an experienced interviewer may have mitigated these limitations. By including both male and female participants from across the Västra Götaland region, providing a thorough description of the analysis process, and using participant quotations to keep the analysis close to the original data, the transferability of the findings is enhanced; however, this must ultimately be judged by the reader.

The current study has important clinical implementations for the further development and implementation of PTR programmes in clinical practice. As mentioned before, home-based rehabilitation or PTR programmes are needed to increase accessibility and remove barriers for participation in exercise programmes for patients with severe lung disease. While PTR has been shown to be effective, it has also been criticised for challenges related to supervision and adherence. It is clear from this qualitative study that technology/exergaming has the potential to guide, support, and facilitate exercise programmes at home, even for elderly patients with severe to very severe pulmonary disease. Nevertheless, adherence to and trust in the system seems to be highly independent on technical stability, availability of technical support, and ease of use. In addition, regular interaction with a supervising physiotherapist emerged as a key component of the programme’s success. In addition, considerable variation was observed in participants’ perceived outcomes, emphasising the need for individualisation and ongoing adaptation of the programme in relation to each patient’s progress. The length and intensity of the programme should therefore be tailored to individual needs, and exergaming technology may not be suitable for all patients.

### Future directions

The current results are encouraging for the further development of gamified PTR technology and provide highly needed insights. Patients should be actively involved in the evaluation of home-based exercise approaches, preferably as partners in a co-design process to advance the methodology. While technology was generally well accepted by participants in the present study, future development should prioritise robustness, stability, and user-friendliness. In addition, the findings indicate that such systems need to be embedded within healthcare programmes that facilitate regular supervision by professional physiotherapists.

## Conclusion

In conclusion, this qualitative interview-based study in patients with COPD who performed a 12-week exergaming based PTR programme revealed the complexity of performing and maintaining exercise in patients suffering from respiratory disease. It showed good potential for technology-supported PTR using exergaming to facilitate the exercise programme, increasing adherence and behavioural change, but also the need for technological robustness, support, and embedment in healthcare programmes.

## Supplementary Information

Supplementary Material 1.

## Data Availability

The datasets generated and/or analysed during the current study are not publicly available due to participant confidentiality and ethical restrictions but are available from the corresponding author on reasonable request and with permission from the Swedish Ethical Review Authority.

## Abbreviations

CAT: COPD Assessment Test
COPD: Chronic Obstructive Pulmonary disease
FEV1: Forced Expiratory Volume in one second
GOLD: Global Initiative for Chronic Obstructive Lung Disease
PR: Pulmonary rehabilitation
PTR: Home-based pulmonary telerehabilitation
RCT: Randomised Controlled Trial
SRQR: Standards of Reporting Qualitative Research
