# Supplementary material for: How do people with COPD experience telerehabilitation using exergaming? A qualitative study
Source: BMC Pulm Med. 2026 Mar 4;26:162. doi: 10.1186/s12890-026-04214-1 (PMC13063678; doi:10.1186/s12890-026-04214-1)
Supplement: Supplementary file 1 — Supplementary Material 1. [file 12890_2026_4214_MOESM1_ESM.docx]

Supplementary file 1

Interview Guide – “Exergaming - Home-monitored pulmonary rehabilitation intervention”

*Purpose of the study:*

*To shed light on experiences and perceptions of training with the Exergaming system for people with chronic obstructive pulmonary disease.*

*The phenomenon: What factors are important to the patient when performing Exergaming?*

**Opening question:**

Can you tell us about your experiences of exercising with the Exergaming system.

**Follow-up questions:**

Tell us about factors that have been important to you in connection with exercising via the system?

(Technology, user-friendliness, training environment (at home), time aspects/availability, education/instruction, the exercises in the training programme, the gaming situation, competing against yourself, feedback from the system)

Did you experience anything that was particularly positive in connection with exercising via the system?

Did you experience any obstacles or concerns in connection with exercising via the system?

(Fear, medical factors, where you trained, the monitoring aspect, competing against yourself)

How do you experience exercise via the system compared to previous training experiences?

How did you experience the role of the physiotherapist in connection with exercising via the system?

(The monitoring aspect, support from the physiotherapist, feedback on the training, adaptation of the training programme, to create motivation)

Now that you have finished training with the system, how do you feel about continuing to exercise?
